# Restoring Anatomical Features in Primary Total Knee Arthroplasty

**DOI:** 10.7759/cureus.40616

**Published:** 2023-06-19

**Authors:** Bogdan Cretu, Mihai Costache, Adrian Cursaru, Bogdan Serban, Razvan Spiridonica, Mihnea Popa, Catalin Cirstoiu, Sergiu Iordache

**Affiliations:** 1 Orthopedics and Traumatology Department, University Emergency Hospital, Bucharest, ROU

**Keywords:** anatomical implant, total, knee, arthroplasty, kinematics

## Abstract

Today, the number of people affected by gonarthrosis symptoms is increasing proportionally. Total knee arthroplasty (TKA) is a successful intervention that aims to reduce pain and restore knee function. However, studies have shown that active young patients still have limitations in performing activities such as skiing, golfing, surfing, and dancing. Over the last few years, total knee arthroplasty has undergone significant changes. Most of the modern TKA implants are designed to reproduce the normal biomechanics of the knee joint, mimicking the physiological pattern with greater compliance in the medial compartment between the tibial insert and femoral condyle and less congruence on the lateral side. Unfortunately, functional outcomes are compromised in approximately half of TKA patients. This loss may be caused by the abnormal kinematics and inherent instability of many contemporary implants. The proper alignment of the femoral component during TKA is a crucial step that influences postoperative results. The position of the femoral component in the axial plane is responsible for flexion stability, knee joint kinematics, flexion alignment, and patellar tracking. The main goal when choosing a type of prosthesis is to achieve an adequate recovery that leads to an improvement in mobility and an increase in the efficiency of the quadriceps.

## Introduction and background

Our patients’ expectations and demands have changed over the years. Patients are much more active; they want to participate in active sports such as skiing, mountain biking, climbing, or racket sports. In the past, traditional total knee replacement (TKR) did not obtain as good results as it does now. In a case-control study, the performance of sports like squatting, golfing, and biking was not as good as expected. Overall, 15% to 20% of knee patients were not satisfied [1-3].

**Figure 1:**
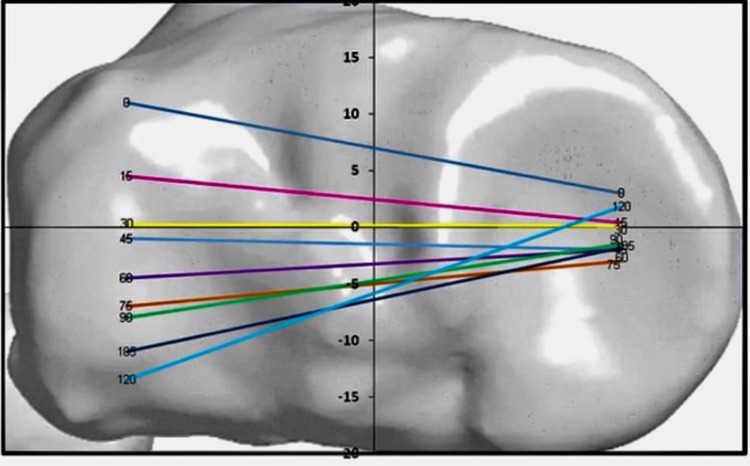
Transverse view of the tibial plateau where we can observe the femur, which is rotating not as a pure medial pivot but more constrained on the medial side than the lateral side

In natural knee kinematics, the femur rotates not as a pure medial pivot but is more constrained on the medial side than the lateral side. All the motion is happening on the lateral side in an anterior-posterior fashion (Figure 1).

In the natural knee, there is no femoral overhanging in full extension, and we have bilateral posterior translation in deep flexion [4-6].

**Figure 2:**
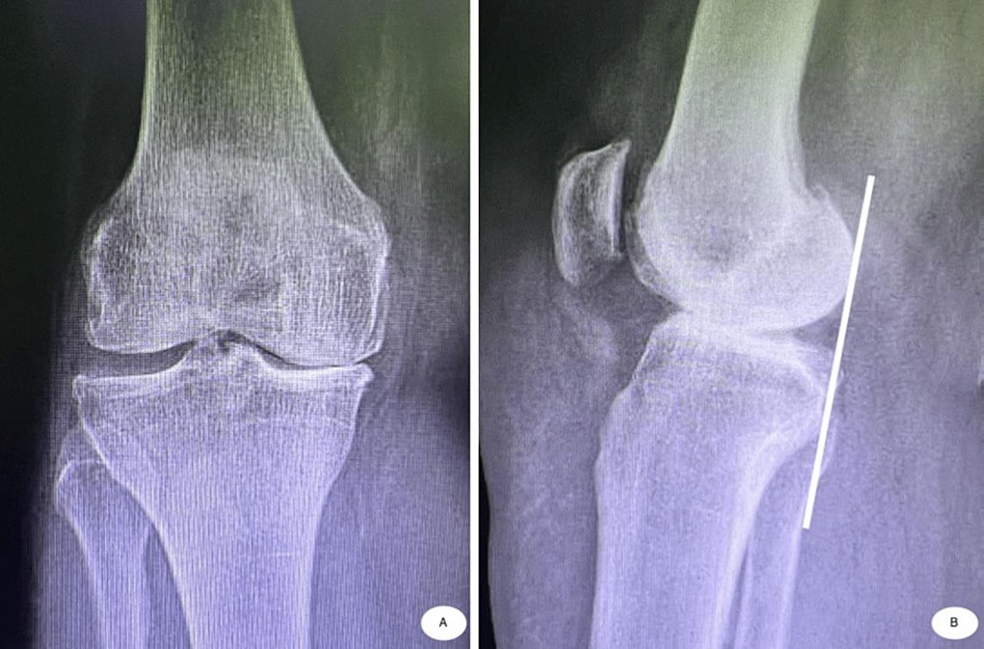
Anterior–posterior (A) and profile view (B) radiograph of the knee before TKA. In the profile view radiograph, we observe a tangent line to the posterior condyles, which passes tangent to the posterior tibial plateau.

**Figure 3:**
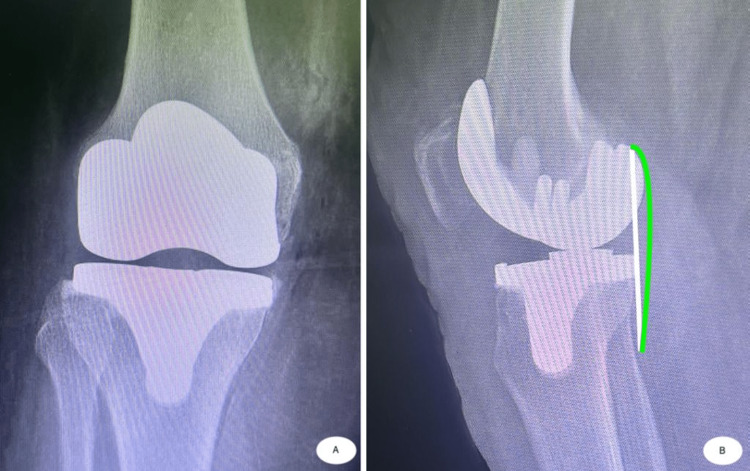
Anterior–posterior (A) and profile view (B) radiograph after TKA. In the profile view radiograph, we observe that after TKA the femur sits posterior to the tibia, and posterior femoral condyles of the prosthesis create pressure on the posterior capsule of the knee.

A traditional total knee replacement shifts anteriorly in early flexion (a paradoxical motion). The femur is in a nonanatomic posterior position, and we have bilateral sliding to create motion. The substituted posterior cruciate ligament (PCL) is replaced by the post-cam mechanism. When we discuss how the conventional knee was designed, we can observe on a profile view radiograph that in a physiological knee, a tangent line to the posterior condyles passes tangent to the posterior aspect of the tibial plateau, whereas in total knee replacement, the femur sits posterior to the tibia (Figures 2-3).

This causes multiple abnormal movements: when the knee flexes, the femur will subluxe forward, creating instability; the ligaments will have no isometry because the femur and the tibia are not lined up; and the muscle function will be abnormal. When the femur sits posteriorly, it artificially tightens the posterior capsule. This is why conventional knee surgical techniques require enough posterior resection or posterior release of the capsule, leading to a very non-anatomical way to reconstruct the knee in many ways, similar to an anterior cruciate ligament (ACL)-deficient knee [7-8].

Abnormal kinematics after total knee arthroplasty (TKA) can be explained by the loss of the ACL and alterations in the surface anatomy after TKA.

As the design of total knee replacements has evolved, issues related to improved mobility as well as postoperative functional capabilities such as stair climbing have become common expectations. Meeting these standards ranged from posterior cruciate ligament retention to posterior cruciate ligament replacement models. The main goal when choosing a type of prosthesis is to achieve an adequate recovery that leads to an improvement in mobility but also an increase in the efficiency of the quadriceps [9]. The argument is that the replacement of the posterior cruciate ligament using a posterior stabilization mechanism is a reliable method of achieving the desired knee kinematics [10,11].

This is a limited narrative review in which we have attempted to highlight certain key aspects of the importance of providing biomechanics in total knee arthroplasty.

## Review

Is there an ideal total knee replacement?

According to studies, they perform the same when patient-recorded outcome measures are evaluated. Many of these studies showed little or no difference between designs despite significant differences in mechanical configuration. The introduction of new and potentially better designs is hampered because large clinical studies are expensive and can take several years. The studies may also be subject to limitations because of the variety of the patients, their comorbidities, and the varying surgical techniques given the subjective ability of the surgeon to perform a TKA [12-14].

Researchers must use objective evaluation methods to decide which TKA performs the best. These could be done by mechanical testing, computer modeling, hybrid methods for laxity measurements, or functional simulations. The criterion to be followed should be the kinematic values of the normal knee. Regarding laxity, we must be aware of the critical difference between testing the laxity of the passive knee and the laxity when we have increased axial compressive force that increases stability [13,14].

Objective Methods to Evaluate Knee Performance

A standard test method for the determination of total knee replacement constraint (ASTM) or computer model of the test: When applied to existing products and proposed prototypes, this test method is intended to produce a database of product functionality capabilities (in light of the suggested test regimens) that would assist the physician in making a more educated TKR selection. A recent study introduced a novel method to evaluate stability in TKA by determining the compressive loading required to achieve natural knee stability. Four current TKA geometries are modeled in a finite-element framework and validated experimentally. Results showed that lower-constraint designs require 66.7% more compressive load than higher-constraint designs to achieve natural knee constraints [15].

A mechanical simulation of the motions of daily living activities: A physical knee model and the virtual ligament hybrid method using an AMTI’s ADL simulator (activities of daily living). It is the most advanced, reliable, and accurate method for evaluating the design and materials of knee implants. It is built to replicate the motions associated with activities of daily living, such as walking and running. Simulators are the most reliable and accurate way to evaluate the design and materials of knee implants.

Computer modeling: Estimates of muscle forces, tibial contact load, location, and pressure distribution can be performed through a combination of mobile fluoroscopy data collection, musculoskeletal modeling, and finite element simulation [16].

When choosing between the variates of TKA regarding restoring normal knee kinematics, we must consider that medial pivot motion in a normally loaded knee leads to medial pivot designs of TKA. Medial anterior-posterior stability involves much more than the lateral side because of the slope of the anterior tibial and the anterior horn of the medial meniscus, which provide resistance to the femur sliding forward [17].

Can bicruciate substituting TKA function similarly to a normal knee?

**Figure 4:**
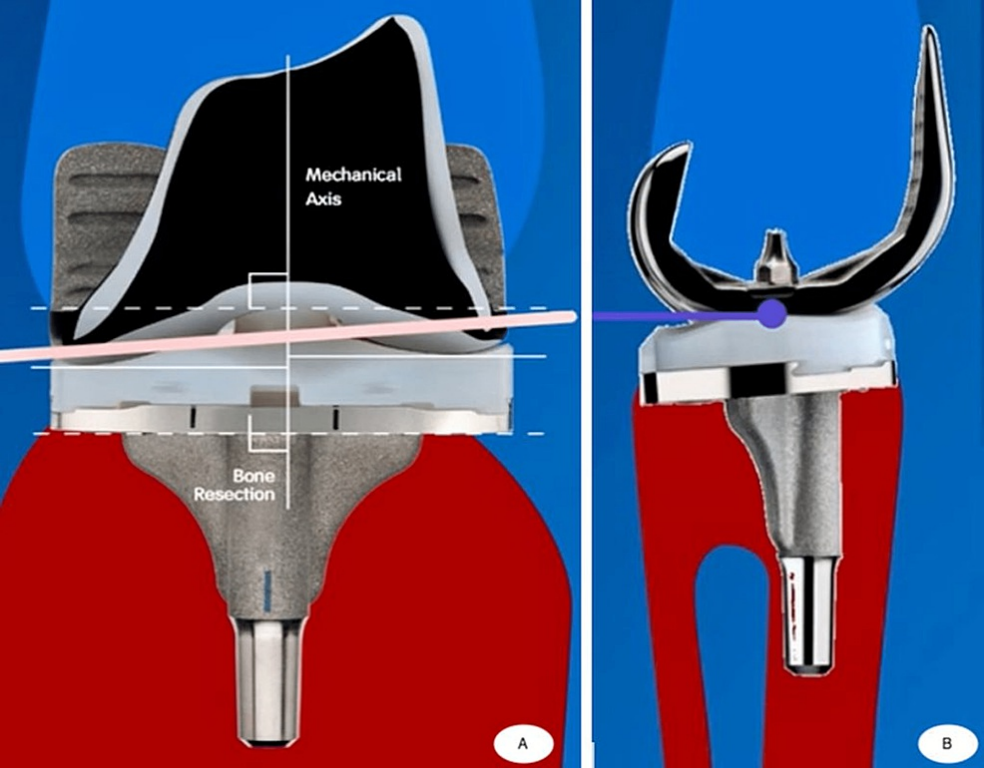
Kinematics total knee implant, where we can observe the three degrees of the anatomic physiological joint line and the obliquity of the tibial plateau (A) and the posterior condyles, which do not pass the posterior tibial plateau (B)

Solutions for overcoming abnormal kinematics include the following: (1) TKA must be viewed as a resurfacing procedure. If our desire is for our patients to move their total knees like normal knees, the implant has to be shaped like the normal knee. (2) The midline sulcus position should be as anatomically correct as possible. The correct position will restore the knee’s normal position, preventing paradoxical motion. It will promote musculature efficiency throughout the range of motion and promote natural patella tracking (Figure 4). (3) Three degrees of the anatomical natural joint line will provide normal ligament tension and natural patella-femoral tracking. (4) The anatomical shape of the prosthetic components - anatomic, asymmetric femur and tibia, and the medial tibial concavity - will promote medial pivot; lateral tibial convexity will promote native rollback because of a sloped lateral compartment [18]. (5) The anterior cam should provide anterior stabilization during early gait (up to 20 degrees of flexion) with a maximized contact area [19-20].

Researchers have suggested that improved TKA kinematics will improve outcomes [19-22]. Clinical studies have also demonstrated short-term safety and effectiveness and improved patient satisfaction scores compared to any other knee included in the studies [23-25]. Snyder et al. found that TKA patients were as satisfied as total hip patients when using a bicruciate-substituting design. This is the first known study to show knee satisfaction rates similar to hip satisfaction [26,27]. The survival of the bicruciate-substituting design was shown to be higher in patients under 55 years old than that of traditional knee replacement, with lower failure rates using this design in both female and male patients [28].

The posterior cruciate ligament - a vestigial organ

Using a posterior cross-substitution design has numerous advantages over cross-retention, including predictable engagement of the post-cam mechanism, predictable range of motion, stability during stair climbing, ease of soft tissue balancing regardless of preoperative deformity, and avoidance of knee problems in which the posterior cruciate ligament is preserved, including posterior cruciate ligament tension or laxity at the time of the procedure and the possibility of late ligament injury, which can lead to joint instability [29].

One of the many debated issues in primary TKA is related to the preservation or sacrifice of the posterior cruciate ligament. There are two main approaches in this case: (1) the anatomical approach of always keeping one or both cruciate ligaments and using bicruciate-retaining or posterior cruciate-retaining implants; and (2) the functional approach to cruciate ligament sacrifice and replacement with posteriorly stabilized implants.

Although there is no consensus, the best clinical outcomes occur when posterior cruciate ligament (PCL) function is retained or restored if resected [30]. Specifically, the implant must allow posterior femoral translation, prevent the femoral condyles from subluxating anteriorly, and regain the anteroposterior stability of the knee. Posteriorly stabilized implants replace the LIP with a post-cam mechanism, but this mechanism is not without complications, which include dislocation and rupture of the polyethylene stabilizer, patellar clunk syndrome and patellar crepitation, distal femoral intercondylar fracture, increased wear around the post, and noise from cam contact and the femoral stabilizer. The ultra-congruent insert was designed as an alternative to the polyethylene stabilizer insert, replacing the LIP but without the aforementioned complications [31].

The ultra-congruent insert, sometimes known as anteriorly stabilized or condylar stabilized, is a polyethylene insert with a large anterior depth and adequate articular surface to prevent anterior subluxation of the condyles. This design replaces the function of the post and thus eliminates post-correlated complications. Because this insert is used with a LIP-retaining femoral component, the need for bone resection from the intercondylar notch to accommodate a posteriorly stabilized femur is avoided, and the procedure requires less operative time, especially when using the tourniquet, which can cause postoperative pain. This makes the ultra-congruent insert an alternative for bone preservation [32]. The ultra-congruent insert is not only a replacement for a posteriorly stabilized femur but also a noteworthy alternative to a standard implant. One of the major difficulties in inserting a cruciate ligament-retaining implant is balancing the soft tissues, but this becomes considerably easier when the LIP is resected. The LIP can also tear or become deficient in a standard cruciate-retaining knee, causing joint instability and the need for revision arthroplasty. Stability is essential in TKA, whether achieved by retaining the LIP or replacing its function with an ultra-congruent or posteriorly stabilized system.

Discussion

From a historical point of view, the restoration of the mechanical axis was considered the gold standard in TKA, with important studies supporting this [33,34]. Several recent factors have cast doubt on mechanical alignment. Biomechanical studies, population studies, and studies that evaluated satisfaction rates all have a common denominator: the restoration of the constitutional axis through kinematic alignment [35-40].

The most important goal that must be achieved is the restoration of ligamentous balance related to the frontal alignment of the TKA as close to normal as physiologically possible so that patients can perform an important series of movements [33]. The same study demonstrated that for an optimal functional result, there is a safe zone of ±2 degrees compared to the constitutional axis so that there is no change in the strained ligament greater than 4% [41-42].

From here derives the discussion of how we can make a TKA with total or partial replacement of the ligaments function as close to normal as possible. It can be deduced that an induced alteration of the collateral ligaments due to the strain leads to problems. Studies have shown that changing the strain by 5.14% will lead to ligamentous structural changes [35]. The statement that constitutional restoration through kinematic alignment is superior to mechanical alignment is supported by statistically significant results [36].

When choosing a TKA system with the goal of restoring normal knee kinematics, we should consider that the medial rotational motion of the normally loaded knee leads to the medial pivot design of the TKA. The medial anteroposterior stability requires much more stability than the lateral because the anterior angle of the anterior tibia and medial meniscus resist sliding the femur forward [17].

## Conclusions

The main goal when choosing a prosthesis type is to achieve adequate recovery, which not only improves mobility but also improves quadriceps performance. The argument is that replacing the posterior cruciate ligament with a posterior stabilization mechanism is a reliable method to achieve desirable knee kinematics. TKA performance can be evaluated with standard tests. They can be of laxity or a simulation of daily living activities, and they can be done by machines or computer models. The reference should be based on normally functioning knees. Recently, the transition from mechanical alignment to kinematic alignment has been attempted, and different knee prostheses are being used to restore the physiological anatomy of the knee as best as possible. The bicruciate-substituting TKA is proven to result in more normal kinematics than the symmetric TKA implant, which does not reproduce the normal kinematics of the physiological knee. Improving TKA kinematics will improve outcomes, such as superior functional outcomes.
